# Insulin Resistance in Systemic Sclerosis: Decoding Its Association with Severe Clinical Phenotype

**DOI:** 10.3390/jcm15020774

**Published:** 2026-01-17

**Authors:** Eugenio Capparelli, Luca Clerici, Giusy Cinzia Moltisanti, Francesco Lapia, Eleonora Zaccara, Francesca Capelli, Daniela Bompane, Maria Sole Chimenti, Sergio Finazzi, Paola Maria Luigia Faggioli, Antonino Mazzone

**Affiliations:** 1 Rheumatology Unit, ASST Ovest Milanese, Legnano Hospital, 20025 Milan, Italy; eleonora.zaccara@asst-ovestmi.it (E.Z.); daniela.bompane@asst-ovestmi.it (D.B.); paola.faggioli@asst-ovestmi.it (P.M.L.F.); 2 Rheumatology, Allergology and Clinical Immunology, Department of Systems Medicine, University of Rome Tor Vergata, 00133 Rome, Italy; maria.sole.chimenti@uniroma2.it; 3 Internal Medicine Unit, ASST Ovest Milanese, Legnano Hospital, Via Papa Giovanni Paolo II, 20025 Milan, Italy; luca.clerici@asst-ovestmi.it (L.C.); giusycinzia.moltisanti@asst-ovestmi.it (G.C.M.); francesco.lapia@asst-ovestmi.it (F.L.); francesca.capelli@asst-ovestmi.it (F.C.); antonino.mazzone@asst-ovestmi.it (A.M.); 4 Department of Internal Medicine and Medical Therapeutics, University of Pavia, 27100 Pavia, Italy; 5 Laboratory of Clinical Chemistry, ASST Ovest Milanese, Hospital of Legnano, 20025 Milan, Italy; sergio.finazzi@asst-ovestmi.it

**Keywords:** insulin resistance, vascular impairment, systemic sclerosis, endothelial dysfunction, metabolic syndrome, cardiovascular events, lung involvement

## Abstract

**Background/Objectives**: Insulin resistance (IR) is a relevant metabolic concern in patients with rheumatic diseases; however, data regarding its clinical influence on the systemic sclerosis (SSc) phenotype is lacking. This study aimed to evaluate the characteristics of patients exhibiting IR in a monocentric SSc cohort. **Methods**: We conducted a cross-sectional study on 178 SSc patients, stratified according to the presence of IR, defined as a HOMA-IR value >1.85 for men and >2.07 for women, based on thresholds previously validated in the *Estudio Epidemiológico de la Insuficiencia Renal en España* (EPIRCE) cross-sectional study. The rationale for applying the current cut-offs is based on its discriminative potential when using sex- and age-specific thresholds in a nondiabetic population. This approach is particularly applicable to SSc, where the prevalence of diabetes is very low and the median ages of the two cohorts are comparable. Data collected included demographic-, clinical-, laboratory-, pulmonary function-, capillaroscopic-, and treatment-related parameters. A multivariable logistic regression model was used to identify independent predictors of IR. **Results**: Patients with IR (*n* = 76) had a significantly higher prevalence of diffuse cutaneous subset (26.3% vs. 11.8%, *p* = 0.012) and interstitial lung disease (39.5% vs. 17.6%, *p* = 0.001), along with the positivity for anti-Scl70 antibodies and the current presence of musculoskeletal symptoms (*p* = 0.021) and digital ulcers (*p* = 0.037). As expected, body mass index (BMI) was significantly higher in the IR population (24.6 ± 5.2 vs. 22.9 ± 4.1, *p* = 0.012), along with fasting glucose, insulin, HOMA-IR, and HbA1c levels. IR patients exhibited higher percentages of dyslipidemia and liver steatosis. Medications such as hydroxychloroquine, statins, and Iloprost were more frequently used in the IR group; as for corticosteroids usage (21.1% vs. 5.9%, *p* = 0.002), however, cumulative glucocorticoid dosage did not differ between the groups. In multivariable analysis, BMI (OR 1.09; *p* = 0.038) and interstitial lung disease (ILD) (OR 3.03; *p* = 0.034) were independent predictors of IR. **Conclusions**: In SSc, IR is associated with ILD, digital ulcers, musculoskeletal involvement, and anti-Scl70 autoantibodies.

## 1. Introduction

Systemic Sclerosis (SSc) is a rare connective tissue disorder marked by microvascular damage, immune system activation, and progressive fibrosis [1]. The dysregulation of the control of the vascular tone, mediated by the endothelium, exerts a pivotal role in the earliest phases of the disease [2,3]. Recent evidence suggests that altered lipid and glucose metabolism, along with a hyperinsulinemic state, can impair endothelial functionality in the general population [4,5,6]. Indeed, at the endothelial level, increased oxidative stress and reduced nitric oxide-mediated vasodilation constitute a shared pathogenic process underlying both chronic insulin resistance and systemic sclerosis-associated vasculopathy [5,7,8].

Insightfully, insulin resistance (IR) is defined as a reduced biological response of target tissues to insulin, occurring primarily in muscle, liver, and adipose tissue. IR is associated with the risk of occurrence of major adverse cardiovascular events (MACE), type II diabetes (T2D), or metabolic syndrome (MetS). However, despite the low prevalence of overt T2D and MetS (7.6% and 10.6%, respectively), the incidence of MACE, cardiac alterations and related mortality is controversially high among patients with SSc, even at the earliest phases of the disease [9,10,11]. Thus, accelerated atherosclerosis and altered reperfusion–ischemia events characterize CV risk excess in this population, and therefore, investigating the relationship between IR and SSc clinical phenotypes could be of interest to rationalize the occurrence of MACE.

IR has also been extensively investigated in autoimmune diseases such as rheumatoid arthritis (RA), systemic lupus erythematosus (SLE), and psoriatic disease (PD). In these conditions, chronic systemic inflammation, glucocorticoid exposure, and reduced physical activity alter adipokine profiles and contribute to the development and persistence of a chronic IR state [12,13,14,15,16,17]. Whether these mechanisms could be applied to metabolic dysfunction related to SSc is challenging. In recent years, glucocorticoid usage has been widely limited due to its established risk to induce scleroderma renal crisis (SRC). Instead, persistent microvascular dysfunction initiates a progressive fibrotic cascade that evolves from endothelial activation and perivascular inflammation to excessive fibroblast activation. In later disease stages, immune system dysregulation appears to have a diminished pathogenic role, distinguishing SSc from other systemic autoimmune rheumatic diseases in which chronic inflammation remains a sustained driver of disease progression, justifying the occurrence of increased dysmetabolic status and CV events [1,18].

No specific studies have been previously oriented to the evaluation of IR nor insulin sensitivity in SSc. Moreover, as previous evidence derives from studies advocating the assessment of both overt MetS and T2DM in this population, the present study aimed to investigate the prevalence and clinical significance of IR in a monocentric cohort of patients with SSc. Specifically, we sought to explore the association of IR with disease-related features, identifying potential independent predictors of IR using multivariate logistic regression analysis, adjusting for known confounders such as age, sex, body mass index (BMI), erythrocyte sedimentation rate (ESR), dyslipidemia, and disease duration.

## 2. Materials and Methods

### 2.1. Study Design and Sample Definition

The present study was a cross-sectional observational study conducted on a monocentric cohort of patients diagnosed with SSc attending the Scleroderma Unit of Legnano Hospital (Azienda Socio Sanitaria Territoriale Ovest Milanese, Legnano, Milan, Italy). The enrollment timeframe was between September 2024 and September 2025. The enrollment period was designed to capture most eligible patients, as most patients are scheduled for follow-up visits every six months. Considering both patients with limited disease and potential missed follow-up referrals, a one-year timeframe was chosen to ensure maximum participation.

Prior to the enrollment, all patients fulfilled the 2013 ACR/EULAR classification criteria for an established diagnosis of SSc [19] and were more than 18 years of age. Patients with an established diagnosis of Type I and II Diabetes Mellitus according to current ADA criteria or patients under antihyperglycemic therapies were excluded from the study [20]. Further exclusion criteria were the presence of MetS according to the National Cholesterol Education Program-Adult Treatment Panel (NCEP-ATP) [21], along with pregnancy state, severe liver and kidney insufficiency, and current antineoplastic treatment or recent diagnosis of any malignancy.

Established cases of MetS and diabetes were specifically excluded from the study to avoid the confounding effects of overt metabolic dysregulation arising from chronic IR. In addition, exclusion of these conditions minimized the potential influence of antidiabetic treatments on insulin and glucose levels, which could have affected the analyses.

The study was approved by the local Ethics Committee (Milan Area 3) and conducted in accordance with the Declaration of Helsinki. All patients were asked to provide signed informed consent to participate in the study.

### 2.2. Assessment of the Presence of Insulin Resistance

To evaluate the presence or absence of IR, blood samples were collected at the enrollment visit, after an overnight fast of at least 8 h. Accordingly, fasting insulin and glucose concentrations were measured using standard chemiluminescent immunoassays by experienced biochemical technicians. The results were confirmed and validated by an experienced clinical pathologist (S.F.). The HOMA-IR index was calculated using the following formula: “HOMA-IR” = (“Fasting Insulin” (μIU/mL) × ”Fasting Glucose” (mg/dL))/405 [22].

Given the lack of a universally accepted and validated cut-off point for the HOMA-IR, IR was defined as a HOMA-IR value > 1.85 for men and > 2.07 for women. These thresholds were adopted from *the Estudio Epidemiológico de la Insuficiencia Renal en España* (EPIRCE) cross-sectional study, which established sex-specific cut-off values based on a large general adult population with demographic and epidemiological characteristics comparable to those of our cohort. The rationale for applying the current cut-offs is based on their discriminative potential when using sex- and age-specific thresholds in a nondiabetic population. These cut-offs have been developed and validated to account for physiological differences related to sex and age, thereby improving the accuracy of insulin resistance assessment in individuals without overt metabolic disease. This approach is particularly appropriate in the context of SSc, which is a condition in which the prevalence of diabetes is very low, and the real prevalence of MetS is currently not well estimated. Moreover, median ages of the two cohorts are comparable. In this context, the selected cut-offs demonstrated good discriminatory ability for identifying individuals with metabolic syndrome, supporting their use as reliable indicators of IR in our study population. Consequently, the use of sex- and age-adjusted cut-offs allows for a more reliable evaluation of subclinical metabolic alterations, minimizing potential bias related to demographic differences between groups [23].

### 2.3. Clinical and Demographic Data Collection

Major disease-specific and patient-related features were collected. To avoid potential confounding effects on IR measurements, demographic and anthropometric data were extracted from medical records and included age at enrollment, age at SSc diagnosis, age at RP onset, sex, smoking status, BMI, body surface area (BSA), and disease duration. Disease duration was defined as the time from the diagnosis of SSc to the last follow-up visit.

Clinical characteristics were collected for all patients at the last follow-up visit, which corresponded to the recruitment date before laboratory analyses were performed. Accordingly, data collected during physical examination comprised (1) the definition of cutaneous subsets, classified according to LeRoy’s criteria (limited, diffuse, or sine scleroderma) [24]; (2) modified Rodnan Skin Score (mRSS) assessment, ranging from 0 to 51 [25]; and (3) the presence or history of RP, digital ulcers, fingertip pitting scars, telangiectasias, tendon friction rubs, gastrointestinal manifestations (including both upper and lower tract symptoms), microcheilia and/or microstomia, calcinosis, sclerodactyly, and musculoskeletal involvement (including the presence of arthralgias, arthritis, myositis and/or myalgias).

The presence of ILD was determined based on the most recent high-resolution computed tomography (HRCT) scan [26]. Pulmonary arterial hypertension (PAH) was defined exclusively by right heart catheterization, and was performed when clinically indicated [27,28,29]. A history or current presence of cardiomyopathy and/or scleroderma renal crisis was retrieved from hospital records. The presence of arrhythmias and left ventricular diastolic dysfunction was assessed using the most recent 24 h electrocardiogram (ECG) and echocardiographic examination, respectively [30,31].

Pulmonary function tests (PFTs) were performed according to ATS/ERS guidelines [32], and the last available assessment reported in medical records was considered for the study. Forced vital capacity (FVC), total lung capacity (TLC), and diffusion capacity for carbon monoxide (DLCO) at single breath (DLCO/SB) and corrected for alveolar volume (DLCO/AV) were expressed as percentages of predicted values. For the analysis, the continuous values expressed as mean ± standard deviation were employed, and no further categorization for abnormal values was made.

NVC examination was performed by trained rheumatologists using a videocapillaroscopy at ×200 magnification. NVC patterns were classified as early, active, or late according to Cutolo’s criteria [33].

Autoantibody profile was extracted from clinical records and included the following: Antinuclear antibodies (ANAs) that were detected by indirect immunofluorescence (IIF) on HEp-2 cell substrates, anticentromere (ACA), anti-topoisomerase I (anti-Scl70), anti-RNA polymerase III, anti-U1-snRNP, anti-Ro/SSA, rheumatoid factor, and other rare SSc-specific antibodies.

Data on comorbid conditions such as arterial hypertension, dyslipidemia, liver steatosis, thyroid disorders, osteoporosis, hyperuricemia, and psychiatric conditions were collected from clinical records. Liver steatosis was assessed by upper abdominal ultrasonography and defined based on the echogenicity criteria, including specifically increased hepatic echogenicity (“bright liver”) compared with the renal cortex.

All imaging procedures, nailfold videocapillaroscopy, and pulmonary function tests were considered at the most recent available assessment within the preceding two years.

Medications including corticosteroids, immunosuppressants, antiplatelet agents, statins, calcium channel blockers, and vasodilators (e.g., Iloprost) were recorded, and were distinguished between ongoing and previous use. Cumulative corticosteroid dose was calculated where available and corresponding mean ± S.D. was included in the analysis as a continuous variable. Moreover, the percentage of patients undergoing current corticosteroid therapy was calculated and included in categorical terms.

### 2.4. Laboratory Assessments

All patients underwent several laboratory test analyses on the same morning of the recruitment day, which included the following:

(1) *Glycated hemoglobin* (HbA1c), measured by high-performance liquid chromatography and related results were expressed as mmol/mol of total hemoglobin and pathological cut-off: ≥48 (diabetes), 39–47 mmol/mol (prediabetes) [34].
(2) *Lipid Profile* comprising total cholesterol (TC), high-density lipoprotein cholesterol (c-HDL), and triglycerides (TG) were measured by enzymatic colorimetric assays. Low-density lipoprotein cholesterol (c-LDL) was calculated using the Friedewald formula (LDL = TC − HDL − [TG/5]) for triglycerides < 400 mg/dL [35]. Reference ranges: Total cholesterol: desirable < 200 mg/dL LDL-C: optimal < 100 mg/dL HDL-C: low < 40 mg/dL (men), <50 mg/dL (women) Triglycerides: normal < 150 mg/dL [36].
(3) *Inflammatory Markers*: C-reactive protein (CRP) expressed in mg/L. Reference range: 0–5 mg/L (normal); ESR with results expressed in mm/hour and reference range: <20 mm/h.
(4) *Renal Function*: Serum creatinine assessment (expressed in mg/dL) and estimated glomerular filtration rate (eGFR), which was calculated using the CKD-EPI Equation (2021) and expressed in mL/min/1.73 m^2^. Pathological cut-off: <60 mL/min/1.73 m^2^ indicates chronic kidney disease (CKD) [37].

### 2.5. Statistical Analysis

Continuous variables were expressed as mean ± standard deviation (S.D.) and compared using Student’s *t*-test or Mann–Whitney U test as appropriate. Categorical variables were expressed as frequencies and percentages and compared using chi-square or Fisher’s exact test, when appropriate. Odds ratios (ORs) with 95% confidence intervals (CIs) were calculated for dichotomous outcomes. A multivariate logistic regression model was constructed to identify independent predictors of IR (defined as dependent variable). The multivariable model was adjusted for several covariates selected based on their recognized clinical and research relevance in both IR and SSc. General covariates included sex, age at enrollment, BMI, ESR, CCS cumulative dosage, and dyslipidemia. Disease-specific variables encompassed disease duration, dcSSc subset, anti-Scl-70 antibody positivity, musculoskeletal involvement, current digital ulcers, the presence of ILD, and FVC% predicted. These variables were included given their well-established association with disease severity and systemic involvement in SSc.

A *p*-value <0.05 was considered statistically significant. Statistical analyses were performed using IBM SPSS Statistics software, version 27 (IBM Corp., Armonk, NY, USA).

## 3. Results

### 3.1. Laboratory Assessment

A total of 76 out of 178 (43%) patients reported a HOMA-IR index greater that employed cut-offs and were accordingly categorized as IR group, while 102 out of 178 (57%) were assigned to no-IR group. Correspondingly, the HOMA-IR index was significantly greater in the IR group (6.6 ± 7.8 vs. 1.2 ± 0.4; *p* < 0.001).

As expected, fasting glucose levels were significantly higher in the IR group compared to the no-IR group (93 ± 12.1 vs. 88.1 ± 9.6 mg/dL; *p* = 0.003), as well for the prevalence of IFG condition [Table 1]. Fasting insulin concentrations were markedly elevated in the IR group (26.8 ± 26.1 vs. 5.4 ± 1.8 µIU/mL; *p* < 0.001), with nearly all IR patients exhibiting insulin levels above 10.57 µIU/mL (94.7% vs. 0%; *p* < 0.001). HbA1c levels were also higher in the IR group compared to no-IR patients (37.7 ± 4.6 vs. 35.7 ± 3.7 mmol/mol), with a significantly lower proportion of IR subjects having HbA1c below 40 mmol/mol (73.7% vs. 89.2%, *p* = 0.006) and a higher proportion with levels ≥48 mmol/mol (5.3% vs. 0%; *p* = 0.032). Lipid profile analysis revealed no significant difference in TC or c-LDL levels between groups. However, c-HDL was significantly lower in the IR group (56.1 ± 16.1 vs. 66.9 ± 16.2 mg/dL, *p* < 0.001), with a higher proportion of IR patients displaying *c*-HDL levels below threshold values (34.2% vs. 11.8%, *p* < 0.001). TG levels were also elevated in the IR group (111.2 ± 50.8 vs. 88.1 ± 38.8 mg/dL, *p* < 0.001), and more IR patients had TG above 130 mg/dL (*p* = 0.012). ESR mean values were marginally higher in the IR group (*p* = 0.029). Renal function was comparable between groups.

### 3.2. Clinical, Demographic, and Anthropometric Characteristics

In the total study population, most patients were female (92.1%), with similar proportions observed in both the IR group (89.5%) and the no-IR group (94.1%) [Table 2].

The mean age at enrollment was 60.6 ± 12.6 years, with no significant difference between groups. Similarly, no significant differences were observed in age at RP onset, age at first non-RP symptom, or age at SSc diagnosis. Disease duration was comparable with an overall mean of 13.3 ± 7.2 years. However, BMI (24.6 ± 5.2 vs. 22.9 ± 4.1 kg/m^2^, *p* = 0.012) and BSA (1.6 ± 0.2 vs. 1.7 ± 0.2 m^2^, *p* = 0.03) were significantly higher in the IR group. Interestingly, current smoking habit was significantly less frequent among IR patients compared to those without IR (10.5% vs. 29.4%, *p* = 0.002).

Regarding skin involvement, the dcSSc subset was significantly more frequent in the IR group (26.3% vs. 11.8%, *p* = 0.012), while no significant differences were observed for lcSSc and sine scleroderma forms. The mRSS was comparable between groups (4.1 ± 4.6 vs. 4.3 ± 3.9, *p* = 0.766), with 14 out 178 patients in the whole cohort exhibiting an mRSS greater than 14 points, with comparable distribution between the two groups.

Regarding the serological profile, the prevalence of ACA was similar between groups (44.7% in IR vs. 49% in no-IR). Conversely, anti-Scl70 antibodies were significantly more frequent in the IR group (28.9% vs. 15.7%, *p* = 0.033). Antibodies against rare SSc antigens were exclusively detected in the IR group (5.3%). Other autoantibodies, including anti-RNA polymerase III, anti-U1-snRNP, anti-RoSSA, and rheumatoid factor, did not differ significantly between groups.

Among clinical signs and symptoms, current digital ulcers, microstomia, and musculoskeletal symptoms were significantly more prevalent in the IR group (*p* = 0.037, *p* = 0.041, *p* = 0.021, respectively), along with ILD that reported 39.5% vs. 17.6% of prevalence compared to no-IR patients, *p* = 0.001. Conversely, PAH occurred only in the no-IR group (5.9%). Other clinical features were evenly distributed between groups. Patients with IR showed a significantly higher prevalence of dyslipidemia (47.4% vs. 17.6%; *p* < 0.001) and liver steatosis (28.9% vs. 11.8%; *p* = 0.004) compared to those without IR. No significant differences were observed between groups for arterial hypertension, thyroid disorders, or other comorbidities.

**Figure 1:**
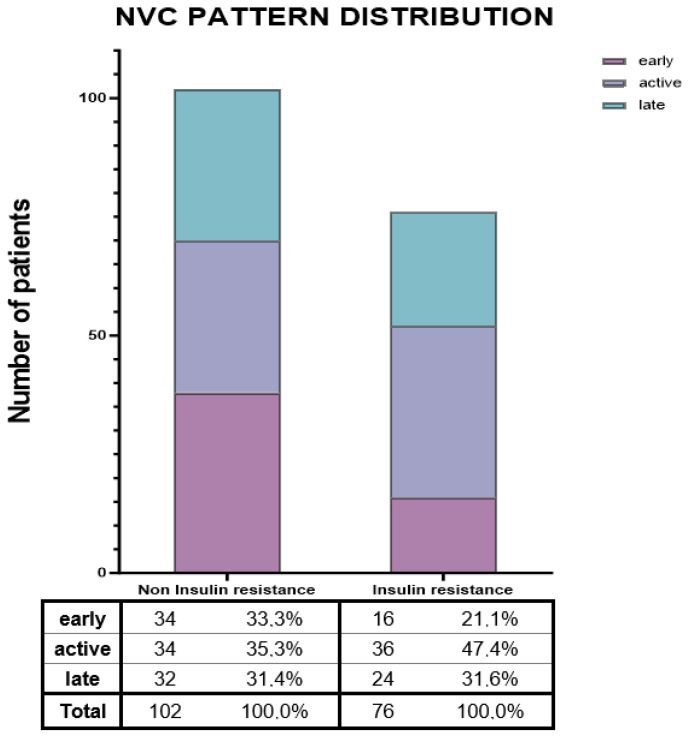
**Nailfold videocapillaroscopic pattern distribution. Abbreviations.** NVC = Nailfold videocapillaroscopy.

Figure 1 revealed a comparable distribution of the late NVC pattern between groups. Early alterations were more frequently observed in the no-IR group, whereas the active pattern was more commonly reported among IR patients (47.4% vs. 35.3%; *p* = 0.057).

**Figure 2:**
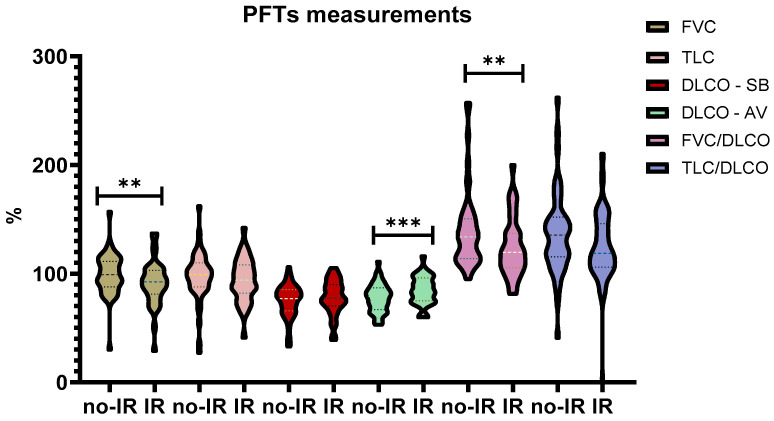
**PFTs measurements comparison. Abbreviations:** PFTs = Pulmonary Function Tests, % = percentages, no-IR = Non-Insulin Resistance, IR = Insulin Resistance, FVC = Forced Vital Capacity, TLC = Total Lung Capacity, DLCO = Diffusing Lung Capacity of Carbon Monoxide. ** = *p* < 0.01, *** = *p* < 0.001.

Regarding PFTs measurements, statistic differences were observed regarding FVC% predicted (91.3 ± 20.7 vs. 99.4 ± 18.7, *p* < 0.01) which was reduced in the IR group, while DLCO/AV% was reduced in the no-IR group (77.5 ± 12.9 vs. 84.6 ± 13, *p* < 0.001). Expectedly FVC/DLCO SB ratio was reduced in the IR group (1.24 ± 0.27 vs. 1.4 ± 0.36, *p* < 0.01) [Figure 2].

### 3.3. Medications Status

Patients with IR were significantly more likely to receive corticosteroid therapy (21.1% vs. 5.9%, *p* = 0.002), although the cumulative dose did not differ significantly between groups [Table 3].

All IR patients were on ongoing Iloprost administrations compared to 88.2% in the no-IR group (*p* = 0.001), with none reporting prior use. Statin or lipid-lowering therapy was also more frequently prescribed among IR patients (28.9% vs. 11.8%, *p* = 0.004). Use of hydroxychloroquine was significantly higher in the IR group (26.3% vs. 7.8%, *p* = 0.0008). While the use of low-dose aspirin was comparable overall, previous use was only reported in the no-IR group. There was more frequent Rituximab use in IR individuals (10.5% vs. 3.9%, *p* = 0.082). No significant differences were observed in the use of calcium channel blockers, mycophenolate mofetil, methotrexate, or cyclophosphamide. ARBs were used more often in the IR group as previous therapy (*p* = 0.032), although ongoing use did not differ significantly. Other immunosuppressive and cardiovascular medications showed similar usage patterns between groups.

### 3.4. Logistic Regression Model

In the binary logistic regression analysis, using the presence of IR above the established cut-off point, BMI was significantly associated with insulin resistance, with higher BMI increasing the odds (adjusted OR 1.09, 95% CI 1.005–1.187, *p* = 0.038) [Table 4].

Similarly, ILD was a significant predictor, showing a threefold increased risk (adjusted OR 3.03, 95% CI 1.086–8.460, *p* = 0.034). Sex, age, disease duration, dcSSc, and anti-Scl-70 antibodies were not significantly associated with IR. There was a trend toward association for musculoskeletal involvement (adjusted OR 2.09, 95% CI 0.911–4.806, *p* = 0.082) and dyslipidemia (adjusted OR 2.09, 95% CI 0.877–4.984, *p* = 0.096). ESR also showed a borderline association (*p* = 0.067). Other variables, including FVC% and corticosteroid cumulative dose, were not significantly related to IR.

## 4. Discussion

IR was notably prevalent in our SSc cohort, affecting 76 out of 178 (43%) of patients, surpassing the pooled prevalence of IR of 26.5% in the adult general population [38]. However, similar findings were reported in established RA cases, where IR prevalence achieved almost 50%, while slightly decreasing in early forms [39,40]. Conversely, the estimated prevalence of IR in SLE was found to be approximately 14% in a study of Quevedo-Abeledo JC, that confirmed a higher prevalence of IR in RA patients compared to SLE [41].

Moreover, in our analysis, the prevalence of IR exceeded previously reported rates of established MetS and T2D in SSc. In a multicenter Italian cohort of 613 patients, the estimated prevalence of T2D was remarkably low, accounting for 7.6% of the population [9], while the real prevalence of MetS in SSc is controversially reported in the literature [10,42,43,44]. For instance, a Mexican study found a prevalence of 36.4%, which is like statistics observed in patients with other systemic autoimmune diseases, but higher than that among the general Mexican population affected by IR [42,43]. Conversely, an Italian study reported a lower prevalence of roughly 19.3% in a cohort of 57 SSc patients. Unlikely, these estimations could be affected by the limited sample size and the presence of genetical and cultural differences between the two populations [44].

Despite the greater prevalence of dyslipidemia and liver steatosis, no relevant differences were noticed on the burden of MACE, BMI values and lipid profiles alterations in the IR group. This suggests that IR in SSc is not merely a marker of metabolic comorbidity but may arise from disease-specific mechanisms such as chronic inflammation, endothelial dysfunction, and altered adipokine signaling.

Beyond its canonical metabolic effects, insulin improves microvascular perfusion and endothelial cell survival and exerts anti-inflammatory and antithrombotic effects [45,46]. Insulin influences immune cell function by modulating cytokine production, leukocyte trafficking, and macrophage polarization in targeted tissues and at vascular beds [47].

In this context, endothelial damage and microvascular rarefaction, typical of SSc, can limit insulin-mediated vasodilation and capillary recruitment, thereby reducing glucose delivery to insulin-sensitive tissues such as skeletal muscle [48]. Simultaneously, chronic low-grade inflammation, a feature of early and acute phases of SSc, is characterized by increased circulating levels of pro-inflammatory cytokines that interfere with insulin receptor signaling [49].

In our analysis, the vascular implications of IR are underscored by its significant association with the presence of active digital ulcers. On the other hand, patients with IR were more likely to display an active capillaroscopic pattern, and as previous evidence related the presence of both enlarged capillaries and microhemorrhages to disease activity in SSc, this data should be interpreted as the presence of increased metabolic demand placed on the endothelium during acute and active disease phases and the association with IR may signal broader systemic metabolic and inflammatory derangement [50].

However, whether IR contributes directly to disease progression or acts as an epiphenomenon of acute mechanisms remains unclear.

Noteworthily, in our study we found a significant association between IR and the presence of ILD, suggesting that IR may be linked to more severe organ involvement. Additionally, the observation of reduced FVC/DLCO ratio, meaning a coherent reduction in both lung volumes and interstitial membrane conductance, typical of fibrotic alteration at parenchymal level in the lungs, aligns with previous studies showing impaired glucose metabolism and worsening lung function in SSc and other fibrotic lung diseases [51,52]. As previously demonstrated from the existence of a metabolic signature in idiopathic pulmonary fibrosis, we could hypothesize that altered insulin metabolic and vascular effects could have exerted a potential role on lung function impairment in IR cases [53]. Accordingly, Jendrek ST et al. showed that patients with SSc-ILD and pulmonary fibrosis exhibit reduced HDL levels in SSc-ILD, alongside the fact that HDL levels correlate with both FVC and DLCO, as well as the mRSS [54]. Reduced HDL levels have been observed in SSs-related PAH cases as a distinct metabolic fingerprint in endothelial cells [55,56]. Cholesterol efflux capacity has been negatively correlated with the extent of skin fibrosis, and a higher monocyte-to-HDL ratio has been linked to digital ulcers, showing a positive correlation with mRSS [54]. In our analysis, the overrepresentation of anti-Scl-70 antibodies and the diffuse cutaneous subset among patients with IR further supports this evidence, adding significance to the hypothesis that dysmetabolic alterations, even at the subclinical level, may be characterized by a more aggressive SSc phenotype.

In the logistic regression analysis, only BMI and ILD emerged as independent predictors of IR, suggesting that the immuno-mediated effect of IR could be a critical marker of SSc-associated ILD. Taken together, these findings highlight the complex interplay between metabolic and pulmonary factors in SSc. Despite the established role of weight loss or reduced BMI in determining worsening of pulmonary fibrosis, recent evidence by Giraudo et al. demonstrated, through a specific analysis of body composition in 87 SSc patients, that both reduced DLCO and higher BMI were independent predictors of mortality. Accordingly, body composition and pulmonary function may independently influence long-term outcomes in SSc and, as we demonstrated, predict IR chronic state [57].

Other clinical features—such as microstomia and musculoskeletal involvement—were also more frequently observed in IR-SSc patients. Similarly, the use of corticosteroids and hydroxychloroquine was higher among individuals with IR. This likely reflects confounding by indication, as patients with more active or inflammatory disease typically require greater glucocorticoid exposure. Conversely, corticosteroid use itself may contribute to disturbances in insulin metabolism, as demonstrated by Atzeni F. et al., who reported higher corticosteroid doses in SSc patients with MetS [44]. Glucocorticoids are well-established triggers of whole-body IR, which exacerbates hepatic steatosis and visceral adiposity, promotes proteolysis and lipolysis in skeletal muscle and adipose tissue, and is associated with β-cell dysfunction [58]. In addition, glucocorticoids have been shown to induce alterations in cardiac function through the development of a chronic insulin-resistant state [59]. This aspect is particularly relevant in SSc, where primary cardiac involvement and myocardial fibrosis are recognized contributors to heart failure and should therefore be carefully considered [60].

Together, these findings indicate that IR may exert a meaningful clinical significance in severe form of SSc. In light of evidence from other autoimmune diseases, targeted modulation of IR, through metformin, GLP-1 receptor agonists, lifestyle interventions, or anti-inflammatory therapies, may represent a promising approach in selected SSc subsets. Of particular relevance is the potential beneficial effect of GLP-1 receptor agonists. This class of glucose-lowering agents has shown to improve insulin sensitivity at the molecular level by enhancing insulin receptor signaling, promoting PI3K/Akt pathway activation, and reducing inflammatory cytokine-mediated interference with insulin action [61]. Clinically, GLP-1 receptor agonists have demonstrated effectiveness in reducing long-term mortality and cardiovascular events in patient affect by rheumatic diseases [62,63]. Their protective effects on the cardiac, renal, and vascular systems, mediated in part through improved insulin sensitivity, may be particularly relevant in SSc.

Several limitations of our study should be acknowledged. The cross-sectional design precludes causal inference. HOMA-IR, while widely used, is an indirect surrogate of insulin sensitivity, and more precise methods (e.g., euglycemic clamp) were not employed. Residual confounding related to diet, physical activity, or sarcopenia cannot be excluded.

A further limitation of this study is the inclusion of multiple covariates in the multivariable model relative to the number of insulin resistance cases, which may increase the risk of overfitting; although these variables were selected a priori based on their established relevance to insulin resistance and disease severity in systemic sclerosis, the findings should be interpreted with appropriate caution.

Finally, the study was monocentric and predominantly female, which may limit generalizability. Accordingly, a multicenter and potentially international cohort would have limited the restriction in the wider applicability of the present results, ultimately acquiring external validity and population-level relevance.

## 5. Conclusions

Our study provides novel evidence that IR is common in SSc, even in the absence of overt T2D or MetS, and may be associated with more severe systemic and vascular manifestations, such as ILD and digital ulcers. These findings highlight the need to consider insulin dysregulation as a disease component in SSc phenotyping.

Prospective studies are required to clarify whether IR represents a distinct clinical subset, acts as a disease modifier, or may serve as a therapeutic target. The longitudinal nature of future studies would clarify the causality between IR and SSc manifestations, ultimately establishing the potential role of IR in disease progression, or conversely, whether IR would be a consequence of the overall disease burden. Addressing metabolic–vascular interactions may open new avenues for personalized management strategies in patients with SSc. Insightfully, the application of both nonpharmacological (e.g., physical activity or patient-oriented dietary approaches) and pharmacological initiatives (e.g., metformin, glucose-lowering therapies) could potentially shape better disease trajectories for SSc patients.

The availability of simple surrogate markers of IR, such as HOMA-IR, despite its indirect methodology for estimating insulin sensitivity, allows for a practical and reproducible assessment of IR in routine clinical and research settings. However, further validation in larger and reproducible cohorts is needed to refine its applicability in clinical practice.

## Figures and Tables

**Table 1:** **Metabolic and laboratory assessment**.

Laboratory Assessment	Total Cohort N = 178	No-IR N = 102	IR N = 76	OR (95% CI)	*p*-Value
Fasting Glucose (mg/dL), mean ± SD	90.2 ± 11.0	88.1 ± 9.6	93 ± 12.1		0.003
IFG, *n* (%)	26 (14.6)	12 (11.8)	14 (18.4)	4.27 (1.58–11.51)	0.214
Fasting Insulin (µIU/mL), mean ± SD	14.6 ± 20.1	5.4 ± 1.8	26.8 ± 26.1		<0.001
Insulin > 10.57 µIU/mL, *n* (%)	72 (40.4)	0 (0)	72 (94.7)	0.04 (0.01–0.10)	<0.001
HOMA-IR, mean ± SD	3.5 ± 5.7	1.2 ± 0.4	6.6 ± 7.8		<0.001
Glycated Hb (mmol/mol), mean ± SD	36.6 ± 4.2	35.7 ±3.7	37.7 ± 4.6		
<40 nmol/mol, *n* (%)	147 (82.6)	91 (89.2)	56 (73.7)	0.34 (0.15–0.76)	0.006
40–47 mmol/mol, *n* (%)	27 (15.2)	11 (10.8)	16 (21.1)	2.21 (0.96–5.08)	0.058
≥48 mmol/mol, *n* (%)	4 (2.2)	0 (0)	4 (5.3)		0.032
Total cholesterol (mg/dL), mean ± SD	193.4 ± 39.8	195.1 ± 39.4	190.1 ± 40.5		0.320
TC > 200 mg/dL, *n* (%)	72 (40.4)	46 (45.1)	26 (34.2)	1.31 (0.91–1.89)	0.143
c-HDL (mg/dL), mean ± SD	62.2 ± 16.9	66.9 ± 16.2	56.1 ± 16.1		<0.001
c-HDL < 40/50 mg/dL, *n* (%)	38 (21.3)	12 (11.8)	26 (34.2)	3.9 (1.81–8.39)	<0.001
c-LDL (mg/dL), mean ± SD	120.5 ± 36.7	120.7 ± 36.9	120.2 ± 36.7		0.930
Triglycerides (mg/dL), mean ± SD	98.2 ± 45.8	88.1 ± 38.8	111.2 ± 50.8		<0.001
TG > 130 mg/dL, *n* (%)	40 (22.5)	16 (15.7)	24 (31.6)	2.48 (1.21–5.10)	0.012
Uric Acid (mg/dL), mean ± SD	4.4 ± 1.1	4.3 ± 1.1	4.6 ± 1		*0.058*
C-Reactive protein (mg/L), mean ± SD	2.5 ± 3.4	2.3 ± 3.3	2.9 ± 3.6		0.281
C-reactive Protein > 5 mg/L, *n* (%)	12 (6.7)	7 (6.9)	5 (6.6)	0.96 (0.29–3.14)	1.0
Erythrocyte Sedimentation Rate (mm/h), mean ± SD	18.6 ± 19.0	15.9 ± 15.8	22.2 ± 22.4		0.029
ESR > 20 mm/h, *n* (%)	44 (24.7)	22 (21.6)	22 (28.9)	1.48 (0.75–2.94)	0.258
CKD-EPI					
e-GFR mL/mix1.73mq, mean ± SD	84.8 ± 19.7	85.7 ± 19.3	83.5 ± 39.4		0.462
Stage 1, *n* (%)	70 (39.3)	42 (42.2)	28 (36.8)	0.83 (0.45–1.53)	0.588
Stage 2, *n* (%)	84 (47.2)	46 (45.1)	38 (50)	1.22 (0.67–2.21)	0.517
Stage 3a, *n* (%)	22 (12.4)	12 (11.8)	10 (13.2)	1.14 (0.46–2.79)	0.779
Stage 3b, *n* (%)	2 (1.1)	2 (2)	0 (0)		0.508

Abbreviations. % = percentages; CKD-EPI = Chronic Kidney Disease Epidemiology Collaboration; CI = Confidence Intervals; c-HDL = high-density lipoprotein cholesterol; c-LDL = low-density lipoprotein cholesterol; dL = deciliters; e-GFR = estimated Glomerular Filtration Rate; ESR = Erythrocyte Sedimentation Rate; HOMA-IR = Homeostatic Model Assessment of Insulin Resistance; IR = Insulin resistance; mg = milligrams; mL = milliliters; mm/h = millimeters/hour; mmol = millimole; mol = mol; N = number; OR = Odds ratio; SD = Standard Deviation; TC = Total Cholesterol; TG = Triglycerides; µIU = micro international unit.

**Table 2:** **Clinical characteristics of the population and comparative analysis between no-IR and IR groups**.

Clinical Characteristics	Total Cohort N = 178	No IR N = 102	IR N = 76	OR (95% CI)	*p*-Value
Sex, *n* (%)					
Female	164 (92.1)	96 (94.1)	68 (89.5)	1.378 (0.845–2.247)	0.255
Male	14 (7.9)	6 (5.9)	8 (10.5)		
Age at enrollment, mean ± S.D.	60.6 ± 12.6	61.4 ± 13.7	59.6 ± 10.8		0.349
Age at RP onset, mean ± S.D.	42.4 ± 15.2	42.3 ± 16.3	42.6 ± 13.7		0.927
Age at 1st non-RP sign/symptom, mean ± S.D.	47.5 ± 13.8	47.9 ± 14.3	46.8 ± 12.9		0.598
Age at SSc Diagnosis, mean ± S.D.	47.3 ± 13.9	48 ± 15.2	46.2 ± 12.2		0.391
Disease Duration, mean ± S.D.	13.3 ± 7.2	13.2 ± 7.2	13.4 ± 7.3		0.841
Body Surface Area, mean ± S.D.	1.7 ± 0.2	1.6 ± 0.2	1.7 ± 0.2		**0.030**
Body Mass Index, mean ± S.D.	23.6 ± 4.7	22.9 ± 4.1	24.6 ± 5.2		**0.012**
Underweight, *n* (%)	20 (11.2)	12 (11.8)	8 (10.5)	0.88 (0.34–2.28)	0.796
Normal weight, *n* (%)	112 (62.9)	66 (64.7)	46 (60.5)	0.84 (0.45–1.54)	0.568
Overweight, *n* (%)	32 (18)	18 (17.6)	14 (18.4)	1.05 (0.49–2.28)	0.894
Obese, *n* (%)	14 (7.9)	6 (5.9)	8 (10.5)	1.88 (0.62–5.67)	0.255
Smoking status, *n* (%)	46 (25.8)	34 (24.1)	12 (17.9)	0.38 (0.18–0.79)	**0.0003**
Current	38 (21.3)	30 (29.4)	8 (10.5)	0.28 (0.12–0.66)	**0.002**
Former	8 (4.5)	4 (3.9)	4 (5.3)	1.36 (0.33–5.62)	0.669
Skin thickening extension, *n* (%)					
Diffuse subtype	32 (18)	12 (11.8)	20 (26.3)	2.68 (1.22–5.9)	**0.012**
Limited subtype	104 (108.4)	62 (60.8)	42 (55.3)	0.8 (0.44–1.46)	0.459
Sine scleroderma	42 (23.6)	28 (27.5)	14 (18.4)	0.6 (0.29–1.23)	0.161
mRSS at last FU, mean ± S.D.	4.2 ± 4.4	4.1 ± 4.6	4.3 ± 3.9		0.766
mRSS > 14, *n* (%)	14 (7.9)	8 (7.8)	6 (7.9)	1.007 (0.33–3.03)	0.990
Serological Profile, *n* (%)					
Anti-centromere	84 (47.2)	50 (49)	34 (44.7)	0.84 (0.47–1.53)	0.571
Anti-Scl70	38 (21.3)	16 (15.7)	22 (28.9)	2.19 (1.06–4.54)	**0.033**
Anti-RNA Polimerase III	2 (1.1)	2 (2.9)	0 (0)	0.57 (0.50–0.65)	0.372
Abs vs. rare SSc antigens	4 (2.2)	0 (0)	4 (5.3)	0.41 (0.35–0.49)	**0.032**
Anti-U1-snRnP	12 (6.7)	6 (5.7)	6 (7.9)	1.37 (0.42–4.43)	0.596
Anti-RoSSA antibodies	22 (12.4)	10 (9.8)	12 (15.8)	1.73 (0.70–4.23)	0.230
Rheumatoid Factor	2 (1.1)	0 (0)	2 (2.6)	0.42 (0.35–0.50)	0.181
Clinical Signs/Symptoms, *n* (%)					
Puffy hands	130 (73)	76 (74.5)	54 (71.1)	0.84 (0.43–1.64)	0.607
Past Digital Ulcers	46 (25.8)	26 (25.5)	10 (26.3)	1.04 (0.53–2.06)	0.901
Current Digitals Ulcers	11 (6.2)	3 (2.9)	8 (10.5)	3.88 (1.0–15.16)	**0.037**
Fingertip pitting scars	62 (348)	38 (37.3)	24 (31.6)	0.78 (0.42–1.46)	0.432
Telangiectasias	80 (44.9)	42 (41.2)	38 (50)	1.43 (0.79–2.59)	0.242
Sclerodactyly	110 (61.8)	64 (62.7)	46 (60.5)	0.91 (0.49–1.67)	0.763
Microstomia	60 (33.7)	28 (27.5)	32 (42.1)	1.92 (1.02–3.61)	**0.041**
Calcinosis	40 (22.5)	22 (21.6)	18 (23.7)	1.13 (0.56–2.29)	0.738
Tendon Friction Rubs	6 (3.4)	2 (2)	4 (5.3)	2.78 (0.49–15.58)	0.227
Musculoskeletal symptoms	67 (37.6)	31 (30.4)	36 (47.4)	2.06 (1.11–3.82)	**0.021**
Upper GI tract involvement	116 (65.2)	62 (60.8)	54 (71.1)	1.58 (0.84–2.98)	0.155
Lower GI tract involvement	48 (27)	26 (25.5)	22 (28.9)	1.19 (0.61–2.31)	0.607
Interstitial lung disease	48 (27)	18 (17.6)	30 (39.5)	3.04 (1.53–6.04)	**0.001**
Pulmonary artery hypertension	6 (3.4)	6 (5.9)	0 (0)	0.56 (0.49–0.64)	**0.031**
Scleroderma renal crisis	6 (3.4)	4 (3.9)	2 (2.6)	0.66 (0.12–3.71)	0.637
Cardiomyopathy	2 (1.1)	0 (0)	2 (2.6)	0.42 (0.35–0.50)	0.099
Arrhythmias	57 (32)	33 (32.3)	25 (32.9)	0.98 (0.52–1.84)	0.939
Left ventricular diastolic dysfunction	65 (36.5)	37 (36.3)	28 (36.8)	1.02 (0.55–1.9)	0.938
Comorbidities, *n* (%)					
Arterial Hypertension	59 (33.1)	29 (28.4)	30 (39.5)	1.64 (0.87–3.08)	0.121
Thyroid disorders	52 (29.2)	31 (30.3)	21 (27.6)	0.87 (0.45–1.69)	0.688
Dyslipidemia	54 (30.3)	18 (17.6)	36 (47.4)	4.2 (2.13–8.3)	**<0.001**
Hyperuricemia	14 (7.9)	6 (5.9)	8 (10.5)	1.88 (0.62–5.67)	0.255
Neurologic issues	14 (7.9)	8 (7.8)	6 (7.9)	1.01 (0.33–3.03)	1.0
Liver steatosis	34 (19.1)	12 (11.8)	22 (28.9)	3.06 (1.4–6.67)	**0.004**
Carotid atherosclerosis	67 (37.6)	37 (36.3)	30 (39.5)	1.15 (0.52–2.11)	0.663
Osteoporosis	67 (37.6)	43 (42.2)	24 (31.6)	0.63(0,34–1.18)	0.150
Previous malignancies	18 (10.1)	12 (11.8)	6 (7.9)	0.64 (0.23–1.8)	0.397
Anxiety/depression	36 (20.2)	22 (21.6)	14 (18.4)	0.82 (0.39–1.12)	0.605

Abbreviations. % = percentage; FU = Follow-up; GI = gastrointestinal; IR = insulin resistance; mRSS = modified Rodnan skin score; N = number; S.D. = standard deviation.

**Table 3:** **Medications**.

Medications, *n* (%)	Total Population N = 178	No IR N = 102	IR N = 76	*p*-Value
Corticosteroids	22 (12.4)	6 (5.9)	16 (21.1)	0.002
Corticosteroids cumulative dosage		0.64 ± 2.16	0.91 ± 1.77	0.351
Iloprost				
Ongoing	166 (93.3)	90 (88.2)	76 (100)	0.001
Previous	8 (4.5)	8 (7.8)	0 (0)	0.011
Low-dose Aspirin				
Ongoing	116 (65.2)	70 (68.6)	46 (60.5)	0.261
Previous	6 (3.4)	6 (5.9)	0 (0)	0.039
Sildenafil				
Ongoing	10 (5.6)	4 (3.9)	6 (7.9)	0.255
Previous	2 (1.1)	2 (2.0)	0 (0)	0.508
ERA				
Ongoing	23 (12.9)	13 (12.7)	10 (13.2)	0.935
Previous	4 (2.2)	2 (2.0)	2 (2.6)	0.765
Calcium Channel Blockers				
Ongoing	50 (28.1)	26 (25.5)	24 (31.6)	0.371
Previous	12 (6.7)	8 (7.8)	4 (5.3)	0.497
Mycophenolate Mophetil				
Ongoing	29 (16.3)	13 (12.7)	16 (21.1)	0.138
Previous		4 (3.9)	6 (7.9)	0.255
Methotrexate				
Ongoing	28 (15.7)	16 (15.7)	12 (15.8)	0.985
Previous	4 (2.2)	2 (1.9)	2 (2.6)	0.765
Cyclophosphamide				
Ongoing	4 (2.2)	2 (2.0)	2 (2.9)	0.765
Previous	10 (5.6)	4 (3.9)	6 (7.9)	0.255
Azathioprine				
Ongoing	6 (3.4)	4 (3.9)	2 (2.6)	0.637
Previous	2 (1.1)	2 (2.0)	0 (0)	0.508
Hydroxychloroquine				
Ongoing	28 (15.9)	8 (7.8)	20 (26.3)	0.0008
Previous	18 (10.2)	8 (7.8)	10 (13.2)	0.244
Tocilizumab				
Ongoing	2 (1.1)	0 (0)	2 (2.6)	0.181
Previ	2 (1.1)	2 (2.0)	0 (0)	0.508
Rituximab				
Ongoing	12 (6.7)	4 (3.9)	8 (10.5)	0.082
Previous	12 (6.7)	4 (3.9)	8 (10.5)	0.082
ARBs				
Ongoing	16 (9)	8 (7.8)	8 (10.5)	0.536
Previous	4 (2.2)	0 (0)	4 (5.3)	0.032
ACEis				
Ongoing	26 (14.6)	14 (13.7)	12 (15.8)	0.680
Previous	0 (0)	0 (0)	0 (0)	/
Beta-blockers				
Ongoing	22 (12.3)	12 (11.8)	10 (13.2)	0.780
Previous	0 (0)	0 (0)	0 (0)	/
Diuretics				
Ongoing	4 (2.2)	4 (3.9)	4 (5.3)	0.497
Previous	0 (0)	0 (0)	0 (0)	/
Statins/lipid lowering therapy				
Ongoing	34 (19.1)	12 (11.8)	22 (28.9)	0.004
Previous	10 (5.6)	7 (6.8)	3 (3.9)	0.403

Abbreviations. N = number; % = percentages; ERA = Endothelin receptor antagonists; ARBs = Angiotensin receptor blockers; ACEis = Angiotensin receptor blockers.

**Table 4:** **Multivariable logistic regression analysis**.

	Beta-Coeff.	Standard Error	Adjusted Odds Ratio	95% CI	*p*-Value
Sex (Female)	−0.912	0.770	0.402	0.089–1.816	0.402
**BMI**	**0.088**	**0.042**	**1.092**	**1.005–1.187**	**0.038**
Age at enrollment	−0.020	0.015	0.177	0.953–1.009	0.981
Disease Duration	−0.035	0.029	0.965	0.913–1.021	0.219
dcSSc	−0.693	0.708	0.500	0.125–2.004	0.328
Anti-Scl70 antibodies	0.201	0.623	1.223	0.361–4.144	0.747
Current Digital Ulcers	0.417	0.765	1.517	0.339–6.797	0.586
Musculoskeletal inv.	0.738	0.424	2.093	0.911–4.806	*0.082*
**ILD**	**1.109**	**0.524**	**3.031**	**1.086–8.460**	**0.034**
FVC%	−0.004	0.011	0.996	0.975–1.017	0.689
ESR (mm/h)	0.020	0.011	1.020	0.999–1.042	*0.067*
CCS cumulative dosage	−0.075	0.109	0.927	0.975–1.147	0.488
Dyslipidemia	0.737	0.443	2.090	0.877–4.984	*0.096*

Abbreviations. BMI = Body mass index; dcSSc = diffuse cutaneous SSc; inv. = involvement; ILD = interstitial lung disease; FVC% = Forced vital capacity; ESR = Erythrocyte sedimentation rate; mm/h = millimeter/hours; CCS = corticosteroids.

## Data Availability

Data will be made available upon reasonable request to the corresponding author, in accordance with privacy regulations and institutional data protection policies.

## Abbreviations

The following abbreviations are used in this manuscript:

ACA	Anticentromere antibodies
ACR/EULAR	American College of Rheumatology/European Alliance of Associations for Rheumatology
ACEi	Angiotensin-converting enzyme inhibitors
ADA	American Diabetes Association
ANA	Antinuclear antibodies
Anti-Ro/SSA	Anti–Sjögren’s-syndrome-related antigen A antibodies
Anti-Scl70	Anti–topoisomerase I antibodies
Anti-U1-snRNP	Anti–U1 small nuclear ribonucleoprotein antibodies
ARBs	Angiotensin II receptor blockers
ATS/ERS	American Thoracic Society/European Respiratory Society
BMI	Body mass index
CCS	Calcium channel blockers
CKD-EPI	Chronic Kidney Disease Epidemiology Collaboration
CRP	C-reactive protein
DLCO	Diffusing capacity for carbon monoxide
eGFR	Estimated glomerular filtration rate
EPIRCE	Estudio Epidemiológico de la Insuficiencia Renal en España
ERA	Early rheumatoid arthritis
ESR	Erythrocyte sedimentation rate
FVC	Forced vital capacity
FU	Follow-up
HbA1c	Glycated hemoglobin
HDL	High-density lipoprotein
HOMA-IR	Homeostatic Model Assessment for Insulin Resistance
HRCT	High-resolution computed tomography
IFG	Impaired fasting glucose
IIF	indirect immunofluorescence
ILD	Interstitial lung disease
IR	Insulin resistance
LDL	Low-density lipoprotein
MACE	Major adverse cardiovascular events
MetS	Metabolic syndrome
mRSS	Modified Rodnan Skin Score
N	Sample size
NCEP-ATP	National Cholesterol Education Program—Adult Treatment Panel
NVC	Nailfold videocapillaroscopy
OR	Odds ratio
PAH	Pulmonary arterial hypertension
PFTs	Pulmonary function tests
RP	Raynaud’s phenomenon
S.D.	Standard deviation
SSc	Systemic sclerosis
T2D	Type 2 diabetes
TC	Total cholesterol
TG	Triglycerides
TLC	Total lung capacity
VEDOSS	Very Early Diagnosis of Systemic Sclerosis
%	Percentage
